# Posterior Ciliary Artery Occlusion Caused by Hyaluronic Acid Injections Into the Forehead: A Case Report

**DOI:** 10.1097/MD.0000000000003124

**Published:** 2016-03-18

**Authors:** Xiu Zhuo Hu, Jun Yan Hu, Peng Sen Wu, Sheng Bo Yu, Don O. Kikkawa, Wei Lu

**Affiliations:** From the Department of Ophthalmology (XZH, JYH, PSW, WL), The Second Hospital of Dalian Medical University, Dalian, Liaoning Province, China; Department of Anatomy (SBY), Dalian Medical University, Dalian, China; Division of Ophthalmic Plastic and Reconstructive Surgery (DOK), UC San Diego Department of Ophthalmology, Shiley Eye Institute, CA.

## Abstract

Although cosmetic facial soft tissue fillers are generally safe and effective, improper injections can lead to devastating and irreversible consequences. We represent the first known case of posterior ciliary artery occlusion caused by hyaluronic acid.

A 41-year-old female presented with right visual loss 7 hours after receiving cosmetic hyaluronic acid injections into her forehead. Examination revealed no light perception in the right eye and multiple dark ischemic area of injection over the forehead and nose. The right fundus revealed a pink retina with optic nerve edema. Fluorescein angiogram showed several filling defects in the choroidal circulation and late hyperfluorescence in the choroid.

A right posterior ciliary artery occlusion and embolic occlusion of facial artery braches was diagnosed. With hyaluronidase injection, hyperbaric oxygen therapy, oral aspirin, oral acetazolamide and dexamethasone venotransfuse treatment, the patient's forehead and nasal skin improved and vision recovered to hand movements.

With proper technique, vascular occlusion is rare following facial filler injection. Vision consequences can be severe if filler emboli enter the ocular circulation. Physicians should be aware of this potential side effect, recognize its presentation, and be knowledgeable of effective management.

## INTRODUCTION

In recent years, cosmetic fillers are becoming increasingly popular for facial rejuvenation, allowing wrinkle reduction, contour improvement, and volume augmentation. Although effective, improper injections can lead to devastating and irreversible consequences. We report a case of blindness caused by soft tissue filler injection into the forehead caused by posterior ciliary artery occlusion.

A retrospective chart review was performed. Institutional review board approval from the Second Hospital of Dalian Medical University was obtained and the tenets of the Declaration of Helsinki were followed.

## CASE REPORT

A 41-year-old woman presented complaining of the sudden visual loss of the right eye while she was receiving the cosmetic forehead filler injection at a beauty salon 7 hours prior. She had no history of prior ocular or systemic disease, and no allergies to medications or known substances. During the procedure, the sudden visual loss was accompanied with severe ocular pain. According to the injector, hyaluronic acid (HA) filler mixed with lidocaine and epinephrine for local anesthesia was used; 2 cm^3^ of HA was injected into the right frontal area with a 23 gauge blunt cannula. During the process of external compression for hemostasis and shaping, the patient abruptly felt sharp pain in her right eye, followed by loss of vision.

**FIGURE 1:**
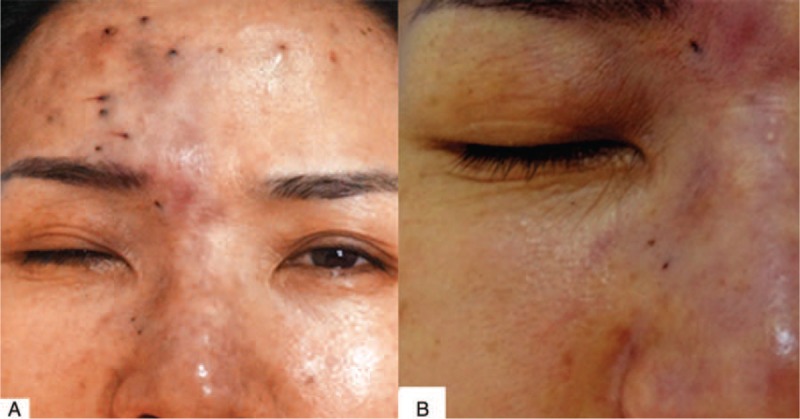
A, A 41-year-old female after soft tissue injections of the face and forehead. Note discoloration of the skin and the presence of several injection points on forehead. B, Note the nasal dorsum skin discoloration and edema.

**FIGURE 2:**
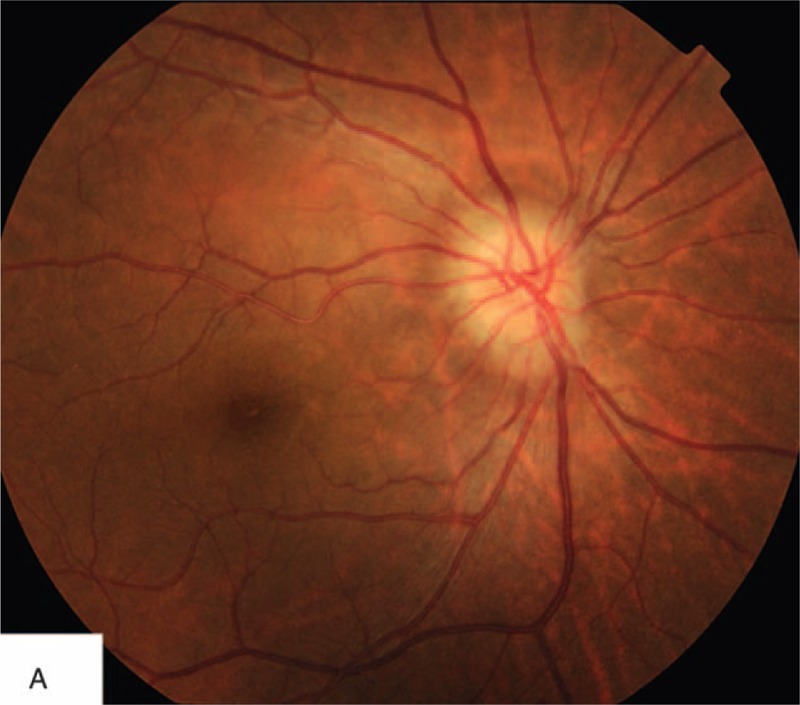
Fundus photograph shows a normal right retina but an edematous optic disc.

**FIGURE 3:**
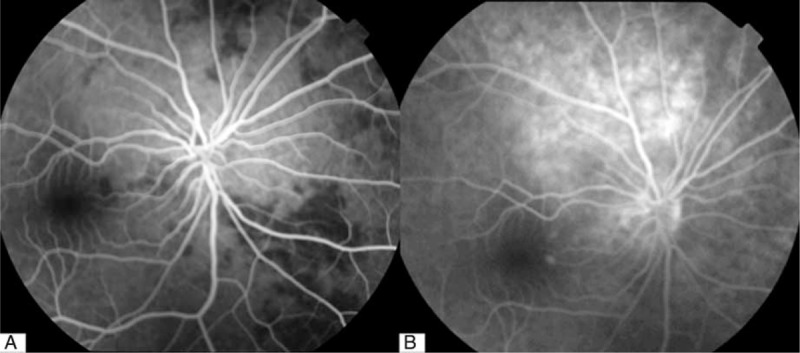
A, Fluorescein angiography of right eye (26.7 seconds after fluorescein injection). Note normal filling of retinal artery but several, fillings in the choroid. B, Fluorescein angiography of right eye (11 minutes and 42 seconds after fluorescein injection). Note late fluorescence leakage from choroid.

**FIGURE 4:**
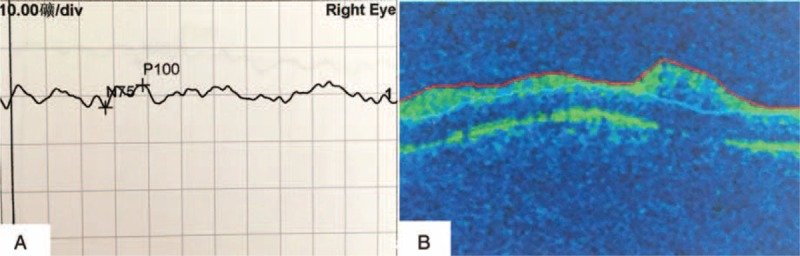
A, Prolonged Wave P100 of the right eye in VEP. B, OCT of the right eye shows a thickened optic disc with normal macula. OCT = optical coherence tomography, VEP = visual evoked potential.

On physical examination, the patient was alert, oriented, and coherent. On the right forehead, there were several areas of dark, the largest measuring about 8 by 5 cm. There were also several injection sites visible. The nasal dorsum skin showed black and purple discoloration with slight edema (Figure 1). Visual acuity was no light perception OD and 20/20 OS. External ocular evaluation showed the right upper eyelid edema and ptosis. A dense right afferent pupillary defect was present. Examination of right fundus revealed a pink fundus with optic nerve edema (Figure 2). A fluorescein angiogram showed normal retinal artery perfusion but several filling defects in the choroidal circulation (Figure 3A). Late hyperfluorescence in the choroid was observed. (T = 11 minutes after fluorescein injection) (Figure 3B). Visual evoked potential was prolonged by P_100_ (Figure 4A). Optical coherence tomography revealed thickening of the optic disc (Figure 4B).

The patient was diagnosed with right posterior ciliary artery occlusion. Packaging material confirmed that the material used was hyaluronic acid. Considering the patient was still in acute period and hyaluronidase was injected into the forehead, glabella, nose, and retrobulbar region (total 1500 U). The patient also received 2 hours of daily hyperbaric oxygen therapy, oral aspirin, oral acetazolamide, and intravenous of dexamethasone were also administered.

**FIGURE 5:**
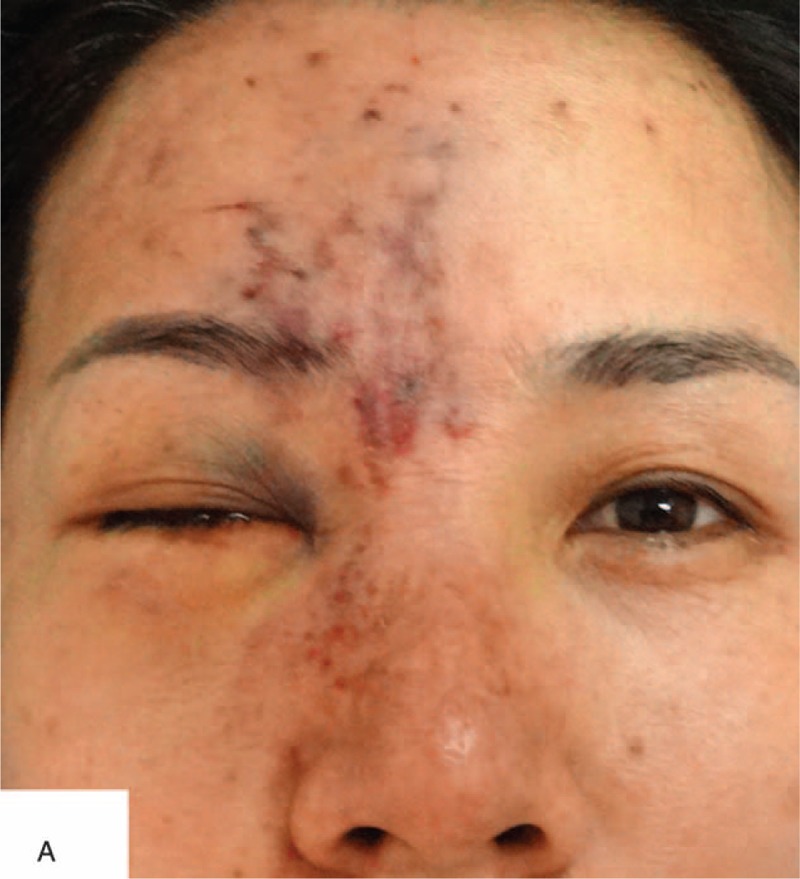
Same patient 2 weeks after treatment. Note improvement in skin discoloration.

At 2-week follow-up of the patient's forehead and nasal skin improved significantly (Figure 5) with improved visual acuity of the right eye to hand movements. Fundus examination revealed decreased edema of the optic disc. After a month of further recovery, the vision was unchanged.

## DISCUSSION

Although HA fillers are generally safe, undesirable effects can occur. Visual complications are the most severe side effects. Intravascular injection is the most devastating complication and can occur with any type of product including autologous material. While our patient was injected in a nonmedical facility, we could not confirm that the procedure was done by a nonmedical professional. Angiogram confirmed posterior ciliary artery occlusion.

Vascular occlusion of any type is an emergency. Periocular vascular events that occur due to injected soft tissue fillers constitute a unique challenge due to the urgent need to restore circulation to the globe to prevent sequelae. Retrograde emboli can enter the ophthalmic artery, central retinal artery or posterior ciliary arteries resulting into retinal ischemia and necrosis, severe ocular pain, vision loss, and other devastating consequences.

**FIGURE 6:**
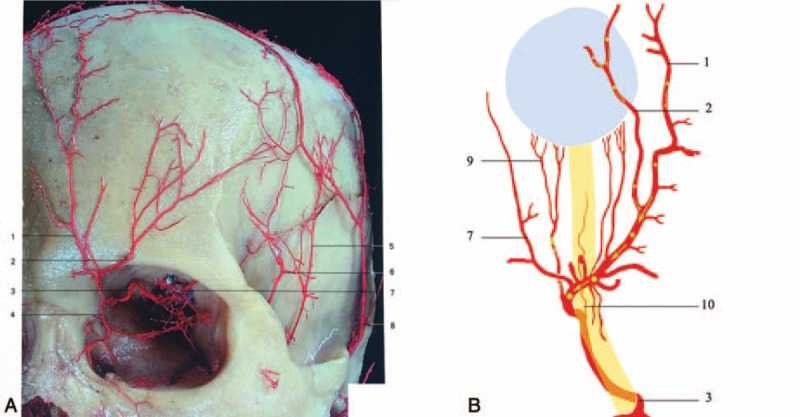
The arteries of peri-orbit. 1—supratrochlear artery, 2—supraorbital artery, 3—continuation of ophthalmic artery, 4—dorsal nasal artery, 5—posterior deep temporal artery, 6—anterior deep temporal artery, 7—lacrimal artery, 8—superficial tempral artery, 9—posterior ciliary artery, 10—central retinal artery.

This case is unique in that it represents the first known case of posterior ciliary artery occlusion caused by hyaluronic acid. In this case, the filler was injected into forehead, glabella, and nose, where known branches of the supraorbital artery, supratrochlear artery, and the dorsal nasal artery exist. To reach the ophthalmic circulation, the concurrent coexistence of 3 factors must occur: the retrograde passage of the material, high injection pressure, and a sufficient amount of material within the vessel lumen.^1^ The force of the injection used for the product delivery can significantly expand arterioles, and injection pressure can be higher than systolic arterial pressure, that would be necessary to retropulse the embolus.^2^ Once reaching theophthalmic artery, the emboli entered the posterior ciliary circulation rather than the central retinal circulation as evidenced by the fluorescein angiogram. The emboli then entered into the chroidal circulation eventually occluded vessels of the choroid (Figure 6).^3^ While external pressure was applied after the injection, it is unlikely to have propagated the emboli.

Injectable fillers can be used in any anatomic region; however, some areas are subject to higher risk of complications. The glabella is the most common site yielding visual complications, followed by the nose, forehead, and periorbital region.^4^ Owing to the abundant terminal blood flow in these areas, the injection technique should consist of slow injections of small amounts of filler during withdrawal of the needle or cannula. Care must be taken to avoid intravascular injections.

In summary, we present the first known case of posterior ciliary artery occlusion casued by hyaluronic acid soft tissue fillers. Extreme caution and care should be given during facial filler injection. Although the incidence is rare, blindness, and permanent visual loss may result.

## References

[1] Egbert JE Paul S Engel WK High injection pressure during intralesional injection of corticosteroids into capillary hemangiomas. *Arch Ophthalmol* 2001; 119:677–683. 11346395 10.1001/archopht.119.5.677

[2] Lazzeri D Agostini T Figus M Blindness following cosmetic injections of the face. *Plast Reconstr Surg* 2012; 129:995–1012. 22456369 10.1097/PRS.0b013e3182442363

[3] Sui HJ Yin L Yu SB Anatomical Altas of Interventional Therapy: Nerves and Blood Vessels. Shenyang: Liaoning Science and Technology Press; 2006.

[4] Ozturk CN Li Y Tung R Complications following injection of soft-tissue fillers. *Aesthet Surg* 2013; 33:862–877. 10.1177/1090820X13493638 23825309

